# The effect of extremely narrow MLC leaf width on the plan quality of VMAT for prostate cancer

**DOI:** 10.1186/s13014-016-0664-0

**Published:** 2016-06-23

**Authors:** Jong Min Park, So-Yeon Park, Jin Ho Kim, Joel Carlson, Jung-in Kim

**Affiliations:** Department of Radiation Oncology, Seoul National University Hospital, Seoul, South Korea; Institute of Radiation Medicine, Seoul National University Medical Research Center, Seoul, South Korea; Biomedical Research Institute, Seoul National University College of Medicine, Seoul, South Korea; Center for Convergence Research on Robotics, Advanced Institutes of Convergence Technology, Suwon, South Korea; Program in Biomedical Radiation Sciences, Department of Transdisciplinary Studies, Seoul National University Graduate School of Convergence Science and Technology, Seoul, South Korea

**Keywords:** Multi-leaf collimator, Leaf width, Volumetric modulated arc therapy, Prostate cancer

## Abstract

**Background:**

To investigate the effect of multi-leaf collimators (MLCs) with leaf width of 1.25 mm on the plan quality of volumetric modulated arc therapy (VMAT) for prostate cancer.

**Methods:**

A total of 20 patients with prostate cancer were retrospectively selected. Using a high definition MLC (HD MLC), primary and boost VMAT plans with two full arcs were generated for each patient (original plan). After that, by shifting the isocenter position of the 2nd arc by 1.25 mm in the cranio-caudal direction, we simulated fluences made with MLCs with leaf width of 1.25 mm. After shifting, primary and boost plans were generated for each patient (shifted plan). A sum plan was generated by summation of the primary and boost plan for each patient. Dose-volumetric parameters were calculated and compared.

**Results:**

Both the homogeneity index (HI) and conformity index (CI) of the shifted plans were better than those of the original plans in primary plans (HI = 0.065 vs. 0.059 with *p* < 0.001 and CI = 1.056 vs. 1.044 with *p* = 0.006). Similarly, the shifted plans for the boost target volume showed better homogeneity and conformity than did the original plans (HI = 0.060 vs. 0.053 with *p* < 0.001 and CI = 1.015 vs. 1.009 with *p* < 0.001). The target mean dose of the original plans was closer to the prescription dose than that of the shifted plans in the case of sum plans (81.45 Gy vs. 81.12 Gy with *p* = 0.001).

**Conclusions:**

Use of extremely narrow MLCs could increase dose homogeneity and conformity of the target volume for prostate VMAT.

## Background

Volumetric modulated arc therapy (VMAT) has been widely adopted in the clinic due to its superior ability to generate optimal dose distributions that deliver prescription doses to target volumes while reducing dose to radiosensitive organs [1–3]. Moreover, VMAT is more efficient in delivery than is intensity modulated radiation therapy (IMRT), spending fewer monitor units (MUs) as well as taking less time to deliver a dose distribution to the patient [4–6]. To acquire the optimal dose distribution, VMAT modulates photon beam intensities by varying multi-leaf collimator (MLC) positions, gantry rotation speed and dose rate, simultaneously, while rotating the gantry around the patient [1, 7–9]. For VMAT, the resolution of the modulated photon beam intensities, i.e. the resolution of fluence, is determined by the MLC leaf width, just as in IMRT [8, 9].

The resolution of the fluence can affect the quality of a plan, which impacts treatment efficacy as well as complications due to radiotherapy. Therefore, several studies have investigated the relationship between MLC leaf width and the quality of IMRT plans [10–12]. They showed that finer leaf widths can result in more conformal dose distributions for target volumes and less dose delivered to normal tissue when the target volumes were small and IMRT was used. Rodal et al. demonstrated that MLCs with fine leaf widths were advantageous for biologically adapted radiotherapy, showing the highest tumor control probability (TCP) and compartmental equivalent uniform dose (EUD) when using the finest MLCs among those they tested, which were MLCs with leaf width of 2.5 mm [11]. Shang et al. demonstrated the dosimetric advantage of utilizing narrow MLC leaves in terms of target volume coverage of prostate IMRT when the treatment was adapted to consider daily prostate movements for simultaneous irradiation of both prostate and pelvic lymph nodes [12]. For VMAT, few studies have investigated the effect of MLC leaf width on the plan quality [10, 13]. Hong et al. have shown the dosimetric advantage of using MLCs with leaf width of 2.5 mm for VMAT for C-shaped head and neck (H&N) cancer by performing a phantom study [10]. Lafond et al. demonstrated that finer MLCs could reduce dose to organs at risk (OARs) for H&N cancer by analyzing a total of 16 VMAT plans [13].

Although several studies have reported the effects of MLC leaf width on the plan quality of VMAT, those studies were performed with currently available MLCs such as the high-definition 120™ MLC (HD 120™ MLC, Varian Medical Systems, Palo Alto, CA), Millennium 120™ MLC (Varian Medical Systems, Palo Alto, CA) and the Beam Modulator™ (Elekta, Stockholm, Sweden) [10, 13]. Therefore, those studies were limited to the leaf width of 2.5 mm, the narrowest leaf commercially available at present. Consequently, the effect of MLC leaf widths narrower than 2.5 mm on VMAT plan quality is unknown. Since it is not clear whether the use of extremely narrow MLCs with leaf widths less than 2.5 mm is advantageous for VMAT plan quality or not, we investigated the impact on plan quality by simulation of virtual MLCs with leaf width of 1.25 mm. Since there is currently no commercially available MLC with 1.25 mm leaf width, we simulated VMAT plans with those MLCs as described below. We generated two full arc VMAT plans with the HD 120™ MLC for prostate cancer, with shifting of the isocenter position of the 2nd arc by 1.25 mm. Although the width of the MLC leaves were still 2.5 mm for each arc, due to VMAT plans consisting of each arc summed together, a 1.25 mm resolution could be simulated through the superposition of two arcs, with a 1.25 mm shift between the arcs. Although this was not a VMAT plan with real MLCs, we assumed that it was a single arc VMAT plan with fluence resolution of 1.25 mm. After simulating the plans, we compared dose-volumetric parameters between the original VMAT plans with two full arcs and no patient shift against the shifted VMAT plans with two full arcs which were assumed to be a single arc VMAT plan with 1.25 mm MLC leaf width. By doing this, we investigated the effect of MLCs with leaf width of 1.25 mm on prostate VMAT plan quality in this study.

## Methods

### Validation of the fine resolution MLC simulation by CT image shift method

To examine the validity of the simulated 1.25 mm width MLCs, created by shifting the isocenter position of the 2nd arc by 1.25 mm using an HD 120™ MLC, we simulated 2.5 mm width MLCs by shifting the isocenter position of a solid water phantom by 2.5 mm with 5 mm width MLCs (Millennium 120™ MLC) in the Eclipse™ system (Varian Medical Systems, Palo Alto, CA). After that, the dose profile of this simulation was compared to that of real 2.5 mm width MLCs (HD 120™ MLC).

For the simulation of 2.5 mm width MLCs by using a CT image shift, one Millennium 120™ MLC in the middle was fully retracted and other MLCs were fully closed. A 10 MV photon beam with 100 MU was delivered to a solid water phantom at a source to surface distance (SSD) of 100 cm. After that, phantom CT images were shifted along the cranio-caudal direction by 2.5 mm and the photon beam with 100 MC was delivered again. A dose profile was analysed along the cranio-caudal direction at a depth of 2.5 cm. For acquisition of the profile with the real 2.5 mm MLCs, the HD 120™ MLC leaf in the middle was fully retracted and other MLC leaves were fully closed. A 10 MV photon beam with 200 MU was delivered to the solid water phantom at 100 cm SSD. After that, only neighbouring upper and lower MLCs from the middle MLC were opened, and a photon beam with 100 MU was delivered. A dose profile was analysed along the cranio-caudal direction at a depth of 2.5 cm. In addition, a dose profile of the 1.25 mm MLC simulation with the same method as the 2.5 mm simulation was acquired with the HD 120™ MLC.

### Patient selection and simulation

A total of 20 patients with prostate cancer who received VMAT in our institution were selected retrospectively for this study. All patients underwent CT scans with a Brilliance CT Big Bore™ (Philips, Cleveland, OH) with slice thickness of 1.5 mm. The resolution of the CT images was 0.98 mm × 0.98 mm (the size of CT voxel = 1.5 × 0.98 × 0.98 mm^3^). Patients were immobilized with a Smart Rest™ (Chunsung, Seoul, Republic of Korea), which is a combination of kneefix and feetfix, in the supine position.

### Generation of original VMAT plans

For each patient, two treatment plans were generated, a plan for a primary target volume (primary plan), and a plan for a boost target volume (boost plan). The primary target volume was delineated with a 2 cm margin in every direction except the posterior and inferior directions from both the prostate and seminal vesicles. To the posterior and inferior directions, 1 cm margins were added in order to reduce dose to the rectal wall. The boost target volume was defined with a 0.7 cm margin in every direction from the prostate. The prescription doses to primary and boost target volumes were 50.4 Gy (daily dose = 1.8 Gy) and 30.6 Gy (daily dose = 1.8 Gy), respectively. Therefore, a total of 81 Gy was delivered to the prostate only, and a total of 50.4 Gy was delivered to the seminal vesicles. Both primary and boost plans were generated with the VMAT technique using the Eclipse™ system. For optimization, the progressive resolution optimizer 3 (PRO3, ver.10, Varian Medical Systems, Palo Alto, CA) was used. The dose volume constraints used during optimization are summarized in Table 1. Initially these constraints were applied identically for every plan, and then the dose volume constraints for OARs were modified by either relaxing or tightening the real-time updated dose-volume histograms (DVHs) as long as it did not harm the quality of the target volumes. By doing this, we accommodated patient-specific differences in the anatomy of each patient. For dose calculation, the anisotropic analytic algorithm (AAA, ver.10, Varian Medical Systems, Palo Alto, CA) was used with a dose calculation grid of 1 mm. For all VMAT plans, the 10 MV photon beam of TrueBeam STx with HD 120™ MLC (Varian Medical Systems, Palo Alto, CA) was used. All VMAT plans were generated with two full arcs. Rectal wall, bladder and femoral heads were defined as OARs. The jaw tracking option was not used and the collimators defining cranio-caudal boundaries (Y jaws) were manually set with a margin of 1 cm from the target volumes. The collimator angle was 0°. The isocenter was determined such that the target volumes were always covered by the inner MLCs of the HD 120™ MLC, of which the leaf width is 2.5 mm. Both primary and boost plans were normalized to cover 97 % of the target volume by at least 97 % of the prescription dose. The sum plans were generated by summation of the primary and boost plans.

**Table 1:** Dose volume constraints for target volumes as well as organs at risk

Structure	Initial dose volume constraints	Relative priority
Primary plan		
Primary PTV	D_100%_ ≥ 99.5 % of the prescription dose	150
	D_98%_ ≥ 100 % of the prescription dose	150
	D_2%_ ≤ 101 % of the prescription dose	150
	D_max_ ≤ 102 % of the prescription dose	150
Rectal wall	V_44Gy_ ≤ 20 %	100
	V_30Gy_ ≤ 50 %	100
Bladder	V_44Gy_ ≤ 20 %	100
	V_30Gy_ ≤ 50 %	100
Femoral heads	V_23Gy_ ≤ 50 %	80
Boost plan		
Boost PTV	D_100%_ ≥ 99.5 % of the prescription dose	150
	D_98%_ ≥ 100 % of the prescription dose	150
	D_2%_ ≤ 101 % of the prescription dose	150
	D_max_ ≤ 102 % of the prescription dose	150
Rectal wall	V_22Gy_ ≤ 20 %	100
	V_18Gy_ ≤ 50 %	100
Bladder	V_22Gy_ ≤ 20 %	100
	V_18Gy_ ≤ 50 %	100
Femoral heads	V_12Gy_ ≤ 50 %	80

*Abbreviations: D*_*n%*_ Dose received by at least *n*% volume of the target volume, *V*_*kGy*_ the percent volume of a structure irradiated by at least k Gy, *PTV* planning target volume

### Simulation of VMAT plans using MLCs with 1.25 mm leaf width (generation of shifted VMAT plans)

To simulate VMAT plans using MLCs with leaf width of 1.25 mm, i.e. half of 2.5 mm, we generated two full arc VMAT plans, and the isocenter position of the 2nd arc was shifted in the inferior direction of the patients by 1.25 mm. Although the width of the MLCs were 2.5 mm for each arc, the resolution of a fluence for a VMAT plan consisting of the two arcs would be 1.25 mm due to the superposition of those two arcs. We assumed those VMAT plans were a single arc VMAT plan with fluence resolution of 1.25 mm (shifted plan). When shifting the isocenter position of the 2nd arc by 1.25 mm, we ensured that the target volumes were fully covered by 2.5 mm MLCs (inner MLCs of HD 120™ MLC). All conditions when generating shifted VMAT plans, including dose-volumetric constraints used during the optimization process, were identical to those of the original VMAT plans with the exception of the shift of the isocenter position of the 2nd arc. Both primary and boost plans were generated and normalized to cover 97 % of the target volume by at least 97 % of the prescription dose, just as in the original VMAT plans. The sum plans were generated by summation of the primary and boost plans.

### Evaluation of dosimetric changes in the shifted VMAT plans compared to the original VMAT plans

To evaluate clinical significance of the fluences with 1.25 mm resolution on the VMAT plan quality, we calculated clinically relevant dose-volumetric parameters for both target volumes and OARs. From each of the primary and boost plans, the mean dose to the target volume, dose received by at least 1 % volume of the target volume (D_1%_), D_99%_, D_95%_, D_5%_, the percent volume of the target volume irradiated by at least 95 % of the prescription dose (V_95%_), *conformity index* (*CI*), *homogeneity index* (*HI*) and *gradient measure* (*GM*) were calculated. The *CI* was calculated as follows [14].

1 $$ Conformity\  index\ (CI)=\frac{Volume\  of\  reference\  isodose}{Volume\  of\  target\  volume} $$

where, the *volume of reference isodose* = volume irradiated by 97 % of the prescription dose since the normalization was performed to cover 97 % of the target volume by at least 97 % of the prescription dose.

The *HI* was calculated as follows [15].

2 $$ Homogeneity\  index\ (HI)=\frac{D_{2\%}-{D}_{98\%}}{D_{50\%}} $$

The *GM* was calculated as follows [16, 17].

3 $$ Gradient\  measure\ (GM)={R}_{50\%\  of\  prescription\  dose}-{R}_{prescription\  dose} $$

where, *R*_*x*_ = the sphere radius of which the volume is the same as the volume of isodose of *x*, and *GM* = an indicator showing the degree of normal tissue irradiation by doses larger than half of the prescription dose.

From the sum plan, the mean dose to the boost target volume and D_1%_, D_99%_, D_95%_ of the boost target volume were calculated. For the primary target volume, the mean dose to the primary target volume and D_99%_, D_95%_ of the primary target volume were calculated. For OARs, mean dose, V_70Gy_, V_47Gy_ and D_50%_ of rectal wall as well as bladder were calculated. For each femoral head, D_50%_ and the maximum dose were calculated. To investigate dose spillage in the normal tissue, V_81Gy_ (100 % of 81Gy), V_72.9Gy_ (90 % of 81Gy), V_64.8Gy_ (80 % of 81Gy), V_50.4Gy_ (100 % of 50.4 Gy), V_45.36Gy_ (90 % of 50.4 Gy), V_40.32Gy_ (80 % of 50.4 Gy) and V_8.1Gy_ (10 % of 81 Gy) of the primary target volume subtracted from the body structure were calculated. To investigate the statistical significance of the differences between the original and shifted VMAT plans, *p* values were calculated using the paired *t*-test, with *p* values less than 0.05 regarded as statistically significant in this study.

## Results

### Validation of the fine resolution MLC simulation by the shift method

**Fig. 1:**
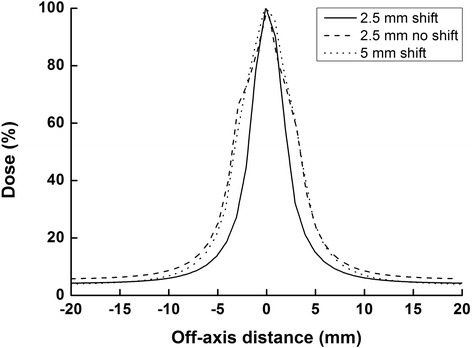
Dose profiles by the simulated and actual multi-leaf collimators (MLCs). Dose profiles of the simulation of 1.25 mm multi-leaf collimator (MLC) with 2.5 mm width MLCs by CT image shift, real 2.5 mm MLCs and the simulation of 2.5 mm MLC with 5 mm width MLCs by CT image shift are plotted with *solid, dashed and dotted lines*, respectively. The profile of the simulation of 2.5 mm MLC was almost identical as that of the real 2.5 mm MLCs

The profiles of the 1.25 mm and 2.5 mm MLC simulations by CT image shift, and the profile of the real 2.5 mm MLCs are plotted in Fig. 1. As shown in Fig. 1, the profile of the 2.5 mm MLC simulation was almost the same as the profile of the real 2.5 mm width MLCs although those profiles were not exactly the same as the design of the HD 120™ MLC (2.5 mm width) is different from that of the Millennium 120™ MLC. The full width at half maximum (FWHM) of the simulation and the real MLCs of 2.5 mm width were 6.782 mm and 6.970 mm, respectively. Therefore, it seems feasible to simulate 1.25 mm MLC by shifting the isocenter position of the 2nd arc by 1.25 mm with HD 120™ MLC. In addition, the FWHM of the 1.25 mm MLC simulation was 4.157 mm.

### Differences in dose-volumetric parameters between original and shifted primary VMAT plans

**Fig. 2:**
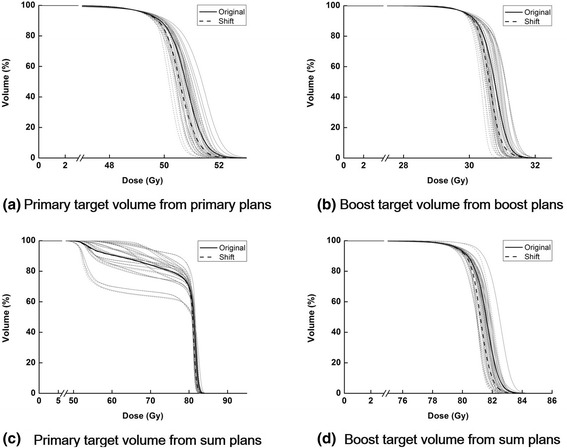
Dose-volume histograms (DVHs) of target volumes. Dose-volume histograms (DVHs) of a primary target volume from primary plans (**a**), boost target volume from boost plans (**b**), primary target volume from sum plans (**c**) and boost target volume from sum plans (**d**) calculated from a total of 20 volumetric modulated arc therapy (VMAT) plans for prostate cancer are shown. The average DVHs of original VMAT plans are plotted with *solid black lines* while those of the shifted VMAT plans are plotted with *dashed black lines*. Every single DVHs of original VMAT plans are plotted with *solid gray lines* while those of the shifted VMAT plans are plotted with *dashed gray lines*

The clinically relevant dose-volumetric parameters of both original and shifted VMAT plans for the primary target volume are shown in Table 2. The average DVH of the primary target volume is calculated from a total of 20 patients and plotted in Fig. 2a. The D_1%_ of the primary target volume of the shifted VMAT plans was lower on average than that of the original VMAT plans with statistical significance (52.0 Gy for original plans vs. 51.7 Gy for shifted plans with *p* < 0.001). The average mean dose to the primary target volume of the shifted VMAT plans was closer to the prescription dose (50.4 Gy) than that of the original VMAT plans (50.7 Gy for original plans vs. 50.5 Gy for shifted plans with *p* = 0.007). The value of D_5%_ of the shifted VMAT plans was lower than that of the original VMAT plans (51.6 Gy for original plans vs. 51.3 Gy for shifted plans with *p* = 0.014). Accordingly, the homogeneity of the dose distribution inside the primary target volume of the shifted VMAT plans was better than that of the original VMAT plans (*HI* = 0.065 for original plans vs. 0.059 for shifted plans with *p* < 0.001). In addition, the conformity of the shifted VMAT plan was better than that of the original VMAT plans (1.056 for original plans vs. 1.044 for shifted plans with *p* = 0.006). However, the power to spare normal tissue by irradiation of intermediate dose (50 % of the prescription dose) of the original VMAT plan was better than that of the shifted VMAT plans (*GM* = 1.86 cm for original plans vs. 2.01 cm for shifted plans with *p* = 0.041). No statistically significant differences in MU were observed between the original and the shifted VMAT plans.

**Table 2:** Average dose-volumetric parameters of primary plans

	Original plan	Shifted plan	*p*
Primary target volume			
D_1%_ (Gy)	51.99 ± 0.36	51.71 ± 0.31	*<0.001*
Mean dose (Gy)	50.70 ± 0.22	50.50 ± 0.20	*0.007*
D_99%_ (Gy)	47.87 ± 0.36	47.94 ± 0.32	*0.026*
D_95%_ (Gy)	49.33 ± 0.08	49.30 ± 0.07	0.107
D_5%_ (Gy)	51.58 ± 0.33	51.32 ± 0.31	*0.014*
V_95%_ (%)	99.07 ± 0.36	99.15 ± 0.35	0.478
Conformity index	1.06 ± 0.05	1.04 ± 0.04	*0.006*
Homogeneity index	0.07 ± 0.01	0.06 ± 0.01	*<0.001*
Normal tissue			
Gradient measure (cm)	1.86 ± 0.18	2.01 ± 0.26	*0.041*
Monitor Unit (MU)			
MU	532 ± 115	572 ± 117	0.294

*Abbreviations: D*_*n%*_ Dose received by at least *n*% volume of the target volume, *V*_*n%*_ the percent volume of a structure irradiated by at least *n*% of the prescription dose

### Differences in dose-volumetric parameters between original and shifted boost VMAT plans

The clinically relevant dose-volumetric parameters of both original and shifted VMAT plans for the boost target volume are shown in Table 3. The average DVH of the boost target volume is plotted in Fig. 2b. Similar results as shown in the primary plans were also observed in the boost plans. The homogeneity of the dose distribution inside the boost target volume was better in the shifted VMAT plans than that of the original VMAT plans (*HI* = 0.060 for original plans vs. 0.053 for shifted plans with *p* < 0.001). Consequently, the value of D_1%_ of the shifted VMAT plans was lower than that of the original VMAT plans, while the opposite was true for the D_99%_, with statistical significance. (D_1%_ = 31.4 Gy for original plans vs. 31.3 Gy for shifted plans with *p* < 0.001 and D_99%_ = 29.2 Gy for original plans vs. 29.3 Gy for shifted plans with *p* < 0.001). The mean dose of the shifted VMAT plans (30.6 Gy) was closer to the prescription dose (30.6 Gy) than that of the original VMAT plans (30.7 Gy) with *p* value of 0.015. Just as the results of the primary plans, conformity of the shifted VMAT plans was better than that of the original VMAT plans (1.015 for original plans vs. 1.009 for shifted plans with *p* < 0.001). However, normal tissue irradiation in the original VMAT plans by intermediate dose was less than that in the shifted VMAT plans with statistical significance. As in the primary plans, no statistically significant differences in MU were observed between the original and shifted VMAT plans.

**Table 3:** Average dose-volumetric parameters of boost plans

	Original plan	Shifted plan	*p*
Boost target volume			
D_1%_ (Gy)	31.43 ± 0.24	31.26 ± 0.31	*<0.001*
Mean dose (Gy)	30.71 ± 0.17	30.57 ± 0.17	*0.015*
D_99%_ (Gy)	29.18 ± 0.10	29.25 ± 0.09	*0.001*
D_95%_ (Gy)	29.92 ± 0.05	29.89 ± 0.04	0.095
D_5%_ (Gy)	31.20 ± 0.22	31.02 ± 0.24	*0.015*
V_95%_ (%)	99.24 ± 0.22	99.38 ± 0.21	*0.039*
Conformity index	1.02 ± 0.02	1.01 ± 0.02	*<0.001*
Homogeneity index	0.06 ± 0.01	0.05 ± 0.01	*<0.001*
Normal tissue			
Gradient measure (cm)	1.66 ± 0.26	1.94 ± 0.47	*0.027*
Monitor Unit (MU)			
MU	470 ± 59	495 ± 61	0.219

*Abbreviations: D*_*n%*_ Dose received by at least *n*% volume of the target volume, *V*_*n%*_ the percent volume of a structure irradiated by at least *n*% of the prescription dose

### Differences in dose-volumetric parameters between original and shifted sum VMAT plans

The clinically relevant dose-volumetric parameters of both original and shifted sum VMAT plans are shown in Table 4. The average DVHs of target volumes of sum VMAT plans are shown in Fig. 2c and d for both original and shifted VMAT plans. For the boost target volume, D_1%_ of the shifted VMAT plans was lower than that of original VMAT plans (83.0 Gy for original plans vs. 82.6 Gy for shifted plans with *p* < 0.001) maintaining same target coverage, which indicates better dose homogeneity in the boost target volume of the shifted VMAT plans than that of the original VMAT plans. The mean dose of the shifted VMAT plans was closer to the prescription dose (81 Gy) than that of the original VMAT plans (81.5 Gy for original plans vs. 81.1 Gy for shifted plans with *p* = 0.001). The value of D_99%_ was higher in the shifted VMAT plans than in the original VMAT plans (78.0 Gy for original plans vs. 78.1 Gy for shifted plans with *p* = 0.014). The dose-volumetric parameters of OARs indicated better plan quality of the shifted VMAT plans than that of the original VMAT plans, showing lower values in the shifted VMAT plans, however, those differences were not statistically significant (all with *p* > 0.05). The value of V_81Gy_ of the whole body showed statistically significant differences between the original and shifted VMAT plans (60.9 cc for original plans vs. 49.0 cc for shifted plans with *p* = 0.007).

**Table 4:** Average dose-volumetric parameters of sum plans

	Original plan	Shifted plan	*p*
Boost target volume			
D_1%_ (Gy)	83.00 ± 0.39	82.55 ± 0.34	*<0.001*
Mean dose (Gy)	81.45 ± 0.32	81.12 ± 0.28	*0.001*
D_99%_ (Gy)	77.98 ± 0.44	78.11 ± 0.37	*0.014*
D_95%_ (Gy)	79.76 ± 0.33	79.68 ± 0.29	0.411
Primary target volume			
Mean dose (Gy)	77.33 ± 2.22	77.04 ± 2.25	0.681
D_99%_ (Gy)	55.11 ± 4.17	55.01 ± 4.23	0.267
D_95%_ (Gy)	59.42 ± 6.27	59.16 ± 6.32	0.896
Rectal wall			
V_70Gy_ (%)	10.56 ± 3.08	10.38 ± 3.08	0.856
V_47Gy_ (%)	32.40 ± 10.04	31.21 ± 9.51	0.700
D_50%_ (Gy)	32.18 ± 11.35	31.22 ± 11.43	0.791
Mean dose (Gy)	36.68 ± 7.05	36.06 ± 6.97	0.783
Bladder			
V_70Gy_ (%)	6.44 ± 4.42	6.37 ± 4.36	0.958
V_47Gy_ (%)	14.35 ± 8.62	14.19 ± 8.51	0.954
D_50%_ (Gy)	12.58 ± 10.11	12.39 ± 10.00	0.952
Mean dose (Gy)	20.62 ± 9.52	20.43 ± 9.46	0.949
Right femoral head			
D_50%_ (Gy)	15.09 ± 2.31	14.88 ± 2.39	0.786
Maximum dose (Gy)	27.51 ± 4.10	27.54 ± 4.44	0.981
Left femoral head			
D_50%_ (Gy)	15.01 ± 3.55	14.79 ± 3.51	0.846
Maximum dose (Gy)	26.68 ± 3.62	27.34 ± 4.65	0.618
Whole body including target volume			
V_81Gy_ (cc)	60.92 ± 14.85	48.96 ± 11.68	*0.007*
Body – primary target volume			
V_81Gy_ (cc)	0.12 ± 0.13	0.07 ± 0.08	0.083
V_72.9Gy_ (cc)	19.23 ± 4.74	18.77 ± 4.59	0.074
V_64.8Gy_ (cc)	43.53 ± 9.61	43.08 ± 9.65	0.290
V_50.4Gy_ (cc)	117.78 ± 23.83	117.06 ± 24.26	0.222
V_45.36Gy_ (cc)	163.60 ± 31.56	163.02 ± 32.28	0.359
V_40.32Gy_ (cc)	226.73 ± 42.88	226.39 ± 44.16	0.707
V_8.1Gy_ (cc)	2963.81 ± 366.10	2979.18 ± 366.93	0.072

*Abbreviations: D*_*n%*_ Dose received by at least *n*% volume of the target volume, *V*_*nGy*_ the percent volume of a structure irradiated by at least n Gy

## Discussion

In this study, we investigated the effect of creating finer resolution fluences on the plan quality of VMAT for prostate cancer by simulating MLCs with 1.25 mm leaf width. The results of both primary and boost plans showed that the dose homogeneity as well as target conformity could be improved by using MLCs with leaf width of 1.25 mm, which was simulated in this study (all with *p* < 0.007). When generating a sum plan, piling up the dose distribution of a boost plan on the homogeneous dose distribution inside a primary target volume can produce high dose conformity to the boost target volume in a sum plan. Therefore, conformity of the boost target volume in a sum plan was much improved by using fine resolution MLCs, while maintaining the same dose coverage of the target volume (V_81Gy_ of normal tissue of the original vs. shifted sum plans = 0.12 cc vs. 0.07 cc with *p* = 0.083). In sequential radiotherapy of a patient, improved dose homogeneity and conformity of the primary and the boost plan induced by the fine resolution MLCs were beneficial for the improving the conformity of the boost target volume in a sum plan. The results showing improved conformity by using fine resolution MLCs were in agreement with the results of previous studies on MLC leaf width [10, 13]. The previous studies also demonstrated that fine resolution MLCs (up to 2.5 mm leaf width) can reduce dose to OARs. In this study, the dose-volumetric parameters for OARs indicated slight reduction of dose by using 1.25 mm MLCs, however, no statistically significant differences between original and the shifted VMAT plans were observed for OARs. For each primary and boost plan, the average values of *GM* of the shifted plans were higher than those of the original plans, which indicated more irradiation by the intermediate dose in the shifted plans. However, the differences were small: 1.5 mm for the primary plans and 2.8 mm for the boost plans. Moreover, the normal tissue volumes irradiated by certain doses (Table 4) of the shifted plans were always smaller than those of the original plans except for V_8.1Gy_. Therefore, no clinically significant differences were observed in normal tissues between the original and the shifted plans in this study. Since the present study was not performed with real 1.25 mm width MLCs but rather performed with the superposition of 2.5 mm width MLCs by shifting the isocenter position of the 2nd arc by 1.25 mm, the degree of freedom of modulation was limited in the present study. For example, if one MLC retracted and delivered 100 MU at a specific control point, the MUs of two neighbouring beamlets (width of beamlet = 1.25 mm) by this MLC were both 100 MUs. Therefore, this limitation of degree of freedom in optimization might cause less improvement in dose-volumetric parameters of OARs and also normal tissues. In addition, the original plans were generated with two full arcs while the shifted plans were generated with a single arc with fluence resolution of 1.25 mm (2 full arcs with 2.5 mm resolution vs. 1 full arc with 1.25 mm resolution). Since previous study has demonstrated that the plan quality of a VMAT plan with multiple arcs is better than that with a single arc, distinguishable improvement in OARs might not be observed in this study [18]. Although 1 full arc was used for the shifted plans, the target conformity and homogeneity were better than those of the original plans with 2 full arcs.

To simulate VMAT plans with 1.25 mm MLC leaf width, we shifted the isocenter position of the 2nd arc. Therefore, we couldn’t evaluate the negative effect of MLCs with leaf width of 1.25 mm, such as increased tongue-and-groove effect or greater interleaf leakage at the leaf junctions. Although this was a limitation of this study, we were able to identify roughly the advantage of MLCs with narrow leaf width on clinically relevant dose-volumetric parameters in this study. This data could serve as a basis as well as a motivation for the development of new MLCs with extremely narrow leaf width.

The dosimetric advantages for OARs in sum plans were observed, although the differences between the original and shifted VMAT plans were not statistically significant. Moreover, MLCs with 1.25 mm leaf width may reduce irradiation of normal tissue by high and intermediate doses in sum plans. The decreased doses to the OARs are meaningful in conjunction with the observed improved dose homogeneity and conformity of the target volume. If we increase the number of samples, statistically significant differences might be acquired. This will be done as a future work.

In theory, MLCs with extremely narrow leaf width can allow a better match of the beam aperture to the target projection, and allow high resolution photon beam optimization with generation of precise field apertures [10]. Consequently, some clinically relevant dose-volumetric parameters, such as dose homogeneity in the target volume, conformity of the prescription dose to the target volume and slight reductions of doses to OARs and reduction of normal tissue irradiation by high dose were improved, while maintaining the same target coverage as conventional MLCs in this study. The improved dose homogeneity of the target volume was beneficial, especially for sequential treatment such as radiotherapy for prostate cancer, since the improved homogeneity of a primary plan induced improved conformity in sum plans. Thus, VMAT plans with extremely fine resolution MLCs might have an important role in tackling challenging prostate cases such as prostate patients with rectal wall very close to the target volume. Moreover, the reduction of high dose irradiation of normal tissue as well as the improvement of conformity of the prescription dose to the target volume by using MLCs with extremely narrow leaf width could be beneficial in many other treatment situations. For example, reducing the maximum dose delivered to OARs located nearby the target volume, such as during stereotactic ablative radiotherapy (SABR) of spine cancer. Although in this study we investigated the dosimetric advantage of fine resolution MLCs only on VMAT plans for prostate cancer, other tumor sites which could take advantage of MLCs with extremely narrow leaf width will be investigated as a future work.

## Conclusions

We investigated the effect of MLCs with extremely narrow leaf widths of 1.25 mm on VMAT plan quality for prostate cancer. We simulated 1.25 mm MLC leaf widths by shifting the isocenter position of the 2nd arc of the VMAT plans using an HD 120™ MLC. The VMAT plans with the simulated fine resolution MLCs showed better dose homogeneity inside the target volume, better target conformity and less dose to normal tissue nearby the target volume, which was irradiated by high and intermediate doses, while maintaining the same target coverage by the prescription dose. It seems that MLCs with extremely narrow leaf width are beneficial during sequential radiotherapy due to the reduction of dose to normal tissue nearby the target volume.

## Ethics approval and consent to participate

Not applicable.

## Consent for publication

Not applicable.

## Availability of data and materials

Our data included the patient information, so we cannot share our planning data.

## Abbreviations

AAA, anisotropic analytic algorithm; CI, aonformity index; D_*n*%,_ dose received by at least *n*% volume of the structure; DVH, dose-volume histogram; EUD, equivalent uniform dose; FWHM, full width at half maximum; GM, gradient measure; H&N, head and neck; HD 120™ MLC, high-definition 120™ multi-leaf collimator; HI, homogeneity index; IMRT, intensity modulated radiation therapy; MLC, multi-leaf collimator; MU, monitor unit; OAR, mrgan at risk; PRO, progressive resolution optimizer; SABR, stereotactic ablative radiotherapy; SSD, source to surface distance; TCP, tumor control probability; VMAT, volumetric modulated arc therapy; V_*n*%,_ volume irradiated by at least *n*% of the prescription dose

## References

[1] Otto K (2008). Volumetric modulated arc therapy: IMRT in a single gantry arc. Med Phys.

[2] Park JM, Kim IH, Ye SJ, Kim K (2014). Evaluation of treatment plans using various treatment techniques for the radiotherapy of cutaneous Kaposi’s sarcoma developed on the skin of feet. J Appl Clin Med Phys.

[3] Park JM, Kim K, Chie EK, Choi CH, Ye SJ, Ha SW (2012). RapidArc vs intensity-modulated radiation therapy for hepatocellular carcinoma: a comparative planning study. Br J Radiol.

[4] Park JM, Wu HG, Kim JH, Carlson JN, Kim K (2015). The effect of MLC speed and acceleration on the plan delivery accuracy of VMAT. Br J Radiol.

[5] Jin H, Jesseph FB, Ahmad S (2014). A comparison study of volumetric modulated Arc therapy quality assurances using portal dosimetry and MapCHECK 2. Prog Med Phys.

[6] Mattes MD, Lee JC, Elnaiem S, Guirguis A, Ikoro NC, Ashamalla H (2014). A predictive model to guide management of the overlap region between target volume and organs at risk in prostate cancer volumetric modulated arc therapy. Radiat Oncol J.

[7] Park JM, Park SY, Kim H, Kim JH, Carlson J, Ye SJ (2014). Modulation indices for volumetric modulated arc therapy. Phys Med Biol.

[8] Park SY, Kim IH, Ye SJ, Carlson J, Park JM (2014). Texture analysis on the fluence map to evaluate the degree of modulation for volumetric modulated arc therapy. Med Phys.

[9] Park SY, Park JM, Sung W, Kim IH, Ye SJ (2015). Texture analysis on the edge-enhanced fluence of VMAT. Radiat Oncol.

[10] Hong CS, Ju SG, Kim M, Kim JI, Kim JM, Suh TS, et al. Dosimetric effects of multileaf collimator leaf width on intensity-modulated radiotherapy for head and neck cancer. Med Phys. 2014;41:021712. 10.1118/1.4860155 24506603

[11] Rodal J, Sovik A, Malinen E (2010). Influence of MLC leaf width on biologically adapted IMRT plans. Acta Oncol.

[12] Shang Q, Qi P, Ferjani S, Xia P (2013). Effect of MLC leaf width on treatment adaptation and accuracy for concurrent irradiation of prostate and pelvic lymph nodes. Med Phys.

[13] Lafond C, Chajon E, Devillers A, Louvel G, Toublanc S, Olivier M, et al. Impact of MLC leaf width on volumetric-modulated arc therapy planning for head and neck cancers. J Appl Clin Med Phys. 2013;14:4074. 10.1120/jacmp.v14i6.4074 PMC5714620 24257269

[14] Park JM, Park SY, Ye SJ, Kim JH, Carlson J, Wu HG (2014). New conformity indices based on the calculation of distances between the target volume and the volume of reference isodose. Br J Radiol.

[15] Prescribing, Recording, and Reporting Photon-Beam Intensity-Modulated Radiation Therapy (IMRT): Contents. *J ICRU* 2010, 10:NP.

[16] Park JM, Choi CH, Ha SW, Ye SJ (2011). The dosimetric effect of mixed-energy IMRT plans for prostate cancer. J Appl Clin Med Phys.

[17] Park JM, Kim JI, Heon Choi C, Chie EK, Kim IH, Ye SJ (2012). Photon energy-modulated radiotherapy: Monte Carlo simulation and treatment planning study. Med Phys.

[18] Tol JP, Dahele M, Slotman BJ, Verbakel WF (2015). Increasing the number of arcs improves head and neck volumetric modulated arc therapy plans. Acta Oncol.

